# Simultaneous Bilateral Anterior Glenohumeral Joint Dislocation: A Case Report

**DOI:** 10.31729/jnma.4949

**Published:** 2020-07-31

**Authors:** Yogendra Agrahari, Marie Joey Lambaco Agrahari, Sangita Karki Kunwor

**Affiliations:** 1 Department of Orthopaedics, Shree Tinau International Hospital, Butwal, Rupandehi, Nepal; 2 Department of Hospice, Access Care Management Consultancy, Van Nuys, California, USA; 3 Department of Global Health and Development, Graduate School of Hanyang University, Seoul, South Korea

**Keywords:** *deformity*, *glenohumeral dislocations*, *immobilization*

## Abstract

The unilateral glenohumeral dislocation is the most commonly encountered dislocation in our practices but the simultaneous bilateral dislocation is very rarely seen entity. It almost always occurs posteriorly. While simultaneous bilateral anterior dislocations present even very rare. We report a case of 70-years-old male who visited to our emergency complex due to trauma after he fell into the bathroom in a drunk state. Patient complains of pain and deformity of both glenohumeral joints. Clinical and radiological findings revealed bilateral anterior glenohumeral joint dislocation. Close reduction under general anaesthesia was done and both shoulders were immobilized using shoulder immobilizer.

## INTRODUCTION

Glenohumeral joint dislocation is one of the most commonly dislocated joints in the human body which reports the incidence of 8.2-23.9 per 100,000 per year.^1^ Shoulder dislocation can be anterior or posterior, unilateral or bilateral. The most commonly encountered is unilateral anterior glenohumeral joint dislocation. Simultaneous bilateral glenohumeral joint dislocation is rarely seen and rarer is anterior type. Bilateral dislocation of the shoulder is often associated with epileptic crises or electrocution and is the most common cause of posterior bilateral dislocations,^2^ while bilateral anterior dislocations are mostly associated with trauma and its occurrence is rarer.^3^ This unusual case report demonstrates a bilateral anterior shoulder dislocation following trauma.

## CASE REPORT

A 70-years old gentleman visited to the emergency complex at 1 am late night with complaints of severe pain (visual analogue score 8/10) and deformity of both shoulders. The patient was in a drunk state and he sustained injury secondary to fall after he slipped in the bathroom with his face forward towards the ground with outstretched hands and both shoulders externally rotated. The past medical history included hypertension with regular follow-up with a cardiologist, with no previous history of trauma, shoulder instability, or epilepsy.

**Figure 1.**
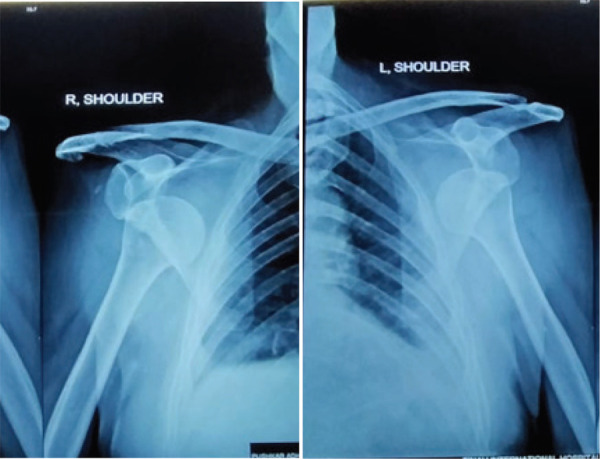
X-ray of bilateral dislocated shoulder.

The physical examination was performed thoroughly in the emergency department, and revealed severe bilateral shoulder tenderness with limited range of motion. The humeral head can be palpable beneath the skin anteriorly, the patient has a limited range of motion in internal rotation and abduction, sulcus sign was positive bilaterally. Patient has lacerated wound approximately 3 × 2 cm at left eyebrow laterally. The neuro-vascular examination revealed normal. No findings of head injury. Standard X-ray of both shoulder joints showed symmetrical antero-medial dislocation without fracture (Figure 1).

**Figure 2.**
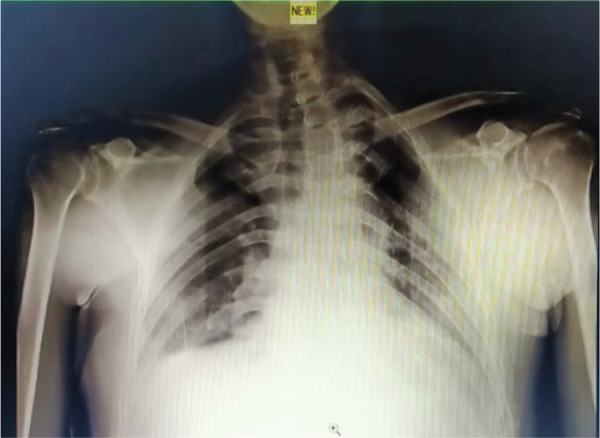
Post close reduction X-ray.

Dislocations were treated under general anaesthesia on the same day by an orthopaedic surgeon. Close reduction by Kocher's technique was done. After close reduction, Dugas test was performed which showed negative. The results of subsequent X-ray imaging showed good anatomical reduction (Figure 2).

**Figure 3.**
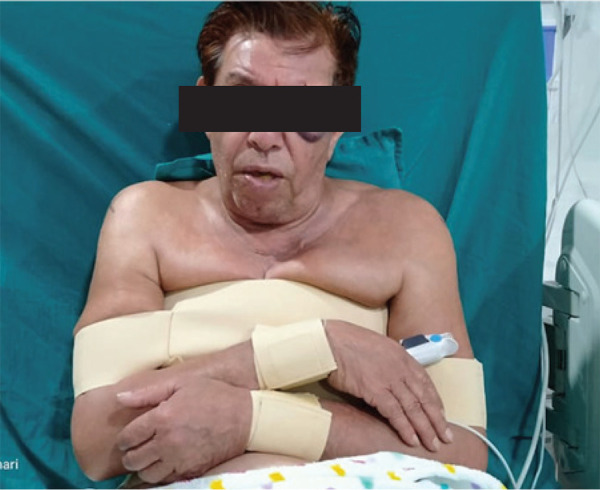
Post reduction and placement of shoulder immobilizer.

Shoulder abduction orthoses were applied bilaterally for 2 weeks then passive to active range of motion were started (Figure 3). Physiotherapy, including shoulder-reconditioning exercises and hot packs, were performed by the physiotherapist as educational session on outpatient basis for a week then patients was advised to continue the exercises at home.

**Figure 4.**
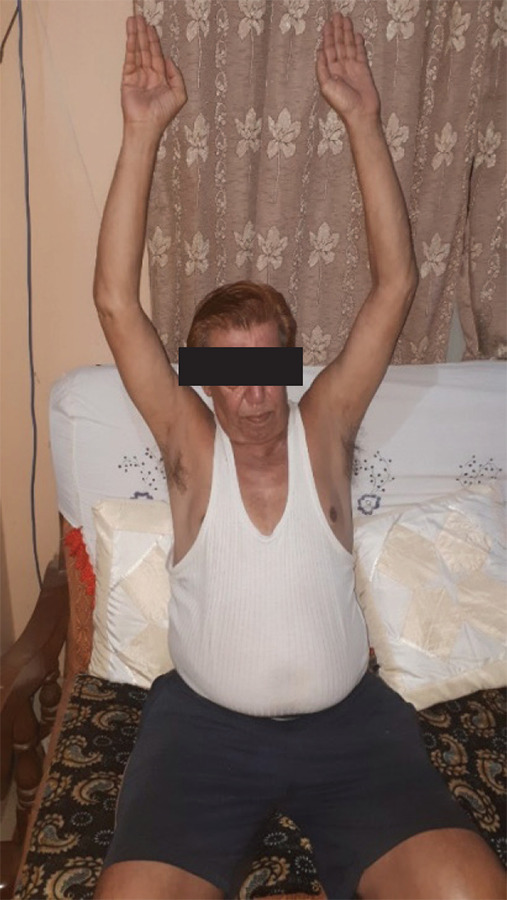
Follow-up on 8 weeks post-reduction.

After four weeks of follow-up, the patient's shoulder pain reduced, visual analogue score was 2/10, and the passive range of motion was nearly full. During 8 weeks follow up, patient have full range of motion with no pain complaints (Figure 4).

## DISCUSSION

Glenohumeral joint dislocation or shoulder joint dislocation is one of the most commonly encountered cases in the emergency department, in contrary simultaneous bilateral shoulder joint dislocation is very rare. Bilateral shoulder joint dislocation was first mentioned in 1902 by Page, et al. about a patient with camphor overdose.^4^ Evidence-based report suggests that bilateral posterior shoulder joint dislocation is rare and however rarer is the bilateral anterior shoulder joint dislocation and few cases are only found in the literature. Most commonly seen bilateral posterior shoulder joint dislocations are due to a sports injury, electrical shock, seizures or hypoglycaemic episodes,^5^ and these occur as a sequel of maximal involuntary muscle contractions while for bilateral anterior shoulder joint dislocation, trauma is the main cause. Our case experienced anterior dislocations of the bilateral glenohumeral joint following a trauma. Several authors have mentioned regarding the bilateral joint dislocation following the trauma of shoulder due to direct impact on the shoulders after fall.^6^ The management principle is same as unilateral glenohumeral joint dislocations during injury and post-reduction that is early reduction and immobilizations which is followed by progressive passive to active exercises.^7^

Bilateral glenohumeral joint dislocations secondary to trauma are very rare cases. This sort of patients should be managed aggressively, diagnosis, and close reduction under sedation should be done at emergency complex if possible, to reduce the pain and long-term complications. Patients need regular follow-up keeping in mind regarding the shoulder instability in future.
